# Engineering for an HPV 9-valent vaccine candidate using genomic constitutive over-expression and low lipopolysaccharide levels in *Escherichia coli* cells

**DOI:** 10.1186/s12934-021-01719-8

**Published:** 2021-12-20

**Authors:** Kaihang Wang, Lizhi Zhou, Tingting Chen, Qiong Li, Jiajia Li, Liqin Liu, Yuqian Li, Jie Sun, Tingting Li, Yingbin Wang, Zhibo Kong, Qingbing Zheng, Jun Zhang, Hai Yu, Ying Gu, Ningshao Xia, Shaowei Li

**Affiliations:** 1 State Key Laboratory of Molecular Vaccinology and Molecular Diagnostics, School of Public Health, School of Life Sciences, Xiamen University, Xiamen, Fujian 361102 China; 2 National Institute of Diagnostics and Vaccine Development in Infectious Disease, Xiamen University, Xiamen, Fujian 361102 China

**Keywords:** *Escherichia coli*, LPS-deficient strain, Chromosomally integrated expression

## Abstract

**Background:**

The various advantages associated with the growth properties of *Escherichia coli* have justified their use in the production of genetically engineered vaccines. However, endotoxin contamination, plasmid vector instability, and the requirement for antibiotic supplementation are frequent bottlenecks in the successful production of recombinant proteins that are safe for industrial-scaled applications. To overcome these drawbacks, we focused on interrupting the expression of several key genes involved in the synthesis of lipopolysaccharide (LPS), an endotoxin frequently responsible for toxicity in recombinant proteins, to eliminate endotoxin contamination and produce better recombinant proteins with *E. coli*.

**Results:**

Of 8 potential target genes associated with LPS synthesis, we successfully constructed 7 LPS biosynthesis-defective recombinant strains to reduce the production of LPS. The endotoxin residue in the protein products from these modified *E. coli* strains were about two orders of magnitude lower than that produced by the wild-type strain. Further, we found that 6 loci—*lpxM, lpxP, lpxL, eptA, gutQ* and *kdsD*—were suitable for chromosomal integrated expression of HPV L1 protein. We found that a single copy of the expression cassette conferred stable expression during long-term antibiotic-free cultivation as compared with the more variable protein production from plasmid-based expression. In large-scale fermentation, we found that recombinant strains bearing 3 to 5 copies of the expression cassette had 1.5- to 2-fold higher overall expression along with lower endotoxin levels as compared with the parental ER2566 strain. Finally, we engineered and constructed 9 recombinant *E. coli* strains for the later production of an HPV 9-valent capsid protein with desirable purity, VLP morphology, and antigenicity.

**Conclusions:**

Reengineering the LPS synthesis loci in the *E. coli* ER2566 strain through chromosomal integration of expression cassettes has potential uses for the production of a 9-valent HPV vaccine candidate, with markedly reduced residual endotoxin levels. Our results offer a new strategy for recombinant *E. coli* strain construction, engineering, and the development of suitable recombinant protein drugs.

**Supplementary Information:**

The online version contains supplementary material available at 10.1186/s12934-021-01719-8.

## Background

*Escherichia coli* (*E. coli*), a rod-shaped Gram-negative bacterium, is an important research platform for synthetic biology [1]. Owing to various advantages, such as low cost, rapid growth, and ease of genetic-manipulation, *E. coli* can be manipulated as a cell factory for the production virus-derived proteins, antibody fragments, enzymes, lipids, and a range of other bio-products [2, 3]. Of particular interest in more recent years is the use of *E. coli* for the manufacture of genetically engineered drugs, such as interferon and insulin [4, 5], and in the development of recombinant human vaccines against hepatitis E virus (HEV) and human papillomavirus (HPV) infection [6, 7].

However, despite these advantages, there are several limitations in using the *E. coli* expression system for the manufacture of exogenous proteins [8, 9]. To date, the synthesis of therapeutic proteins using the *E. coli* expression system at a laboratory scale have been based on plasmid expression, with an antibiotic resistance gene usually incorporated into the plasmid for cloning and stability. However, in large-scale fermentation, the structural and segregational instability of plasmids and the metabolic burden on the cells for high rates of plasmid replication have a negative impact on gene expression fidelity and host strain viability [10]. Additionally, the use of antibiotics carries considerable risk for public health and the environment [11] and thus must be excluded in the culture. Such high-density continuous fermentation using antibiotic-free culture carries inherent problems with batch consistency [12]. Finally, an additional concern with Gram-negative bacteria is the extra-membrane component, lipopolysaccharide (LPS), an endotoxin that, in high concentrations, induces pyretic response and septic shock in mammalian hosts [13]. Therapeutic proteins expressed using the *E. coli* expression system can be easily contaminated with LPS and, thus, residual endotoxin contamination in recombinant therapeutic proteins is strictly monitored [14]. As yet, there is no efficient method to remove LPS completely[15].

To overcome these drawbacks, one solution is to integrate heterologous genes into the chromosomes of prokaryotic hosts for stable expression. Indeed, this is a routine approach for the over-expression of recombinant proteins in eukaryotic cell systems, such as the CHO system for antibody production [16]. Many loci on the *E. coli* chromosome are appropriate insertion sites for exogenous genes, and several recombinant *E. coli* strains have been constructed for the production of valuable bio-products via the establishment of novel synthesis pathways [17–19]. Previous research suggests that there are three main factors to consider when constructing a chromosomally integrated expression strain. First, the integration site(s) on the *E. coli* chromosome should be transcriptionally active and accessible to RNA polymerase [20–22]. Second, the heterologous protein and its coding sequences that are to be introduced should not be lethal to the bacterial host cells [23]. And third, the copy number of the inserted genes should be considered, as this can affect the expression efficiency [24].

Previous work sought to construct an endotoxin-free *E. coli* strain by identifying and editing genes that were non-essential for growth (*lpxL*, *lpxM*, *lpxP*, *eptA*, *pagP*, *kdsD, msbA* and *gutQ*) that also correlated with the LPS synthesis pathway [25–28]. Upon silencing these genes, LPS was converted into lipid IV_A_ and anchored outside the membrane of the recombinant strain. This resultant endotoxin precursor lipid IV_A_ showed no specific immune response in vitro [29].

Building on these findings, the aim of the present study was to construct an *E. coli* germline for the stable expression of virus capsid proteins with low intrinsic endotoxicity for human recombinant vaccine production by disrupting LPS-associated genes. We first investigated the transcriptional activity of the loci that serves the LPS synthesis pathway through strand-specific transcriptomic sequencing. The expression cassettes for the L1 proteins from different HPV genotypes were, respectively, inserted into the loci using the CRISPR-Cas9 genome editing system in combination with lambda-Red recombination system. We then compared plasmid-based L1 expression with chromosomally integrated L1 expression prepared at the shake-flask level and in large-scale fermentation. Moreover, we investigated the effect of copy number on L1 production from the integrated expression cassettes. Finally, we constructed 9 recombinant *E. coli* strains for L1 proteins of HPV types 6, 11, 16, 18, 31, 33, 45, 52, 58, and demonstrated desirable purity, VLP morphology and antigenicity of the various L1 proteins.

## Results

### Construction and validation of the LPS synthesis-defective *E. coli* ER2566 strain

**Fig. 1:**
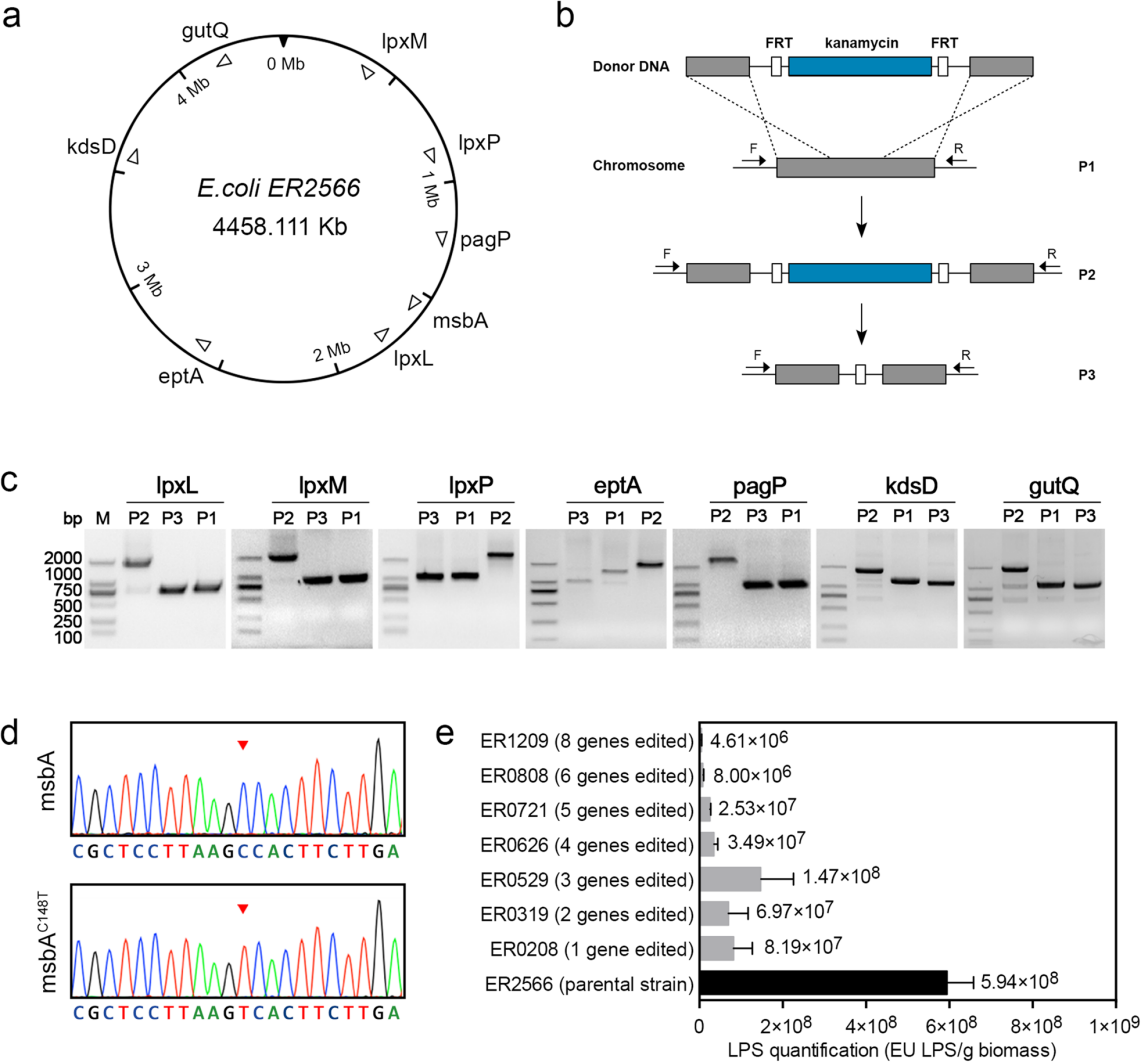
Construction and evaluation of an LPS-defective *E. coli* ER2566 strain. **a** Schematic map of the chromosome of the *E. coli* strain, ER2566. Location and transcriptional orientation of the genes associated with LPS synthesis are indicated by open arrows. **b** Insertion of the donor DNA into the bacterial chromosome via homologous recombination. The solid arrows marked with “F” and “R” indicate the forward and reverse primers, respectively, used for PCR verification. **c** PCR analysis of the resultant *E. coli* strains at each genome editing step. Lanes marked with “M” indicate the DNA marker. Lanes marked with “P1”, “P2” and “P3” indicate the PCR products of target genes. **d** DNA sequencing of the *msbA* gene. The nucleotide mutant (from C to T) is indicated by the red arrow. **e** Endotoxin unit equivalents of the recombinant *E. coli* strains as determined by TAL assay

We first sought to construct an *E. coli* strain that conferred chromosomally integrated expression and that was defective in endotoxin synthesis. Eight endogenous genes—*lpxL*, *lpxM*, *lpxP*, *eptA*, *pagP*, *kdsD*, *msbA* and *gutQ*—involved in the synthesis, modification and transportation of the LPS molecule, but not essential for growth, were targeted and edited sequentially. The distribution and transcriptional orientation of these genes are shown in Fig. 1a. For gene editing, we inserted into the target gene a linear donor DNA containing an FRT-flanked kanamycin expression cassette with 300~400 bp homology extensions on both sides for colony screening (lambda RED recombinase). The kanamycin selection marker was then later excised by flippase-mediated recombination, leaving the single FRT site and part of the target gene deleted (Fig. 1b). As shown in Fig. 1c, the PCR products of the edited genes (*lpxL*, *lpxM*, *lpxP*, *eptA*, *pagP*, *kdsD* and *gutQ*) were about 2000 bp after insertion of the antibiotic selection marker (lane P2), decreasing to ~1000 bp when the antibiotic selection marker was excised (lane P3). Correct recombination was confirmed by DNA sequencing (data not shown). To generate a single nucleotide mutation (in *msbA* at position 148 from C to T) CRISPR/Cas9 was used in cooperation with the homology-directed repair (Fig. 1d). The constructed *E. coli* strains are summarized in Table 1.

**Table.1:** Summary of the constructed *E. coli* strains

Strains	Relevant genotype	Source or reference
ER2566	F^−^ λ^−^ fhuA2 [lon] ompT lacZ::T7 gene 1 gal sulA11 Δ(mcrC-mrr)114::IS10 R(mcr-73::miniTn10-Tet^S^)2 R(zgb-210::Tn10)(Tet^S^) endA1 [dcm]	Laboratory strain
ER0208	ER2566(lpxL::FRT)	This study
ER0319	ER0208(lpxM::FRT)	This study
ER0529	ER0319(lpxP::FRT)	This study
ER0626	ER0529(eptA::FRT)	This study
ER0721	ER0626(pagP::FRT)	This study
ER0808	ER0721(kdsD::FRT)	This study
ER1209	ER0808(msbAC148T)(gutQ::FRT)	This study

Next, the LPS produced by these modified *E. coli* strains was quantified and compared with that of the parental ER2566 strain. The bacterial was harvested and subsequently disrupted by supersonic, and the supernatant of the cell lysate were detected by Tachypleus amebocyte lysate (TAL) assay as a measurement of the LPS concentration. For the genome-modified strains, the determined endotoxin unit (EU) equivalents ranged from 1.47 × 10^8^ EU/g to 4.61 × 10^6^ EU/g, with the level decreasing against an increasing number of modified genes (Fig. 1e). The parental *E. coli* strain ER2566, had EU equivalents of 5.94 × 10^8^ EU/g, which is approximately 4- to 100-fold higher than that of the genome-modified strains. These data indicate that modifying the genes associated with LPS synthesis could significantly decrease the residual amount of endotoxin in the cell lysates. The resultant strains, particularly ER0808 and ER1209, with 6 and 8 edited genes, respectively, exhibited the lowest endotoxin levels as compared with the parental strain.

### Evaluation of LPS synthesis-correlated loci for chromosomally integrated expression of the HPV capsid protein

**Fig. 2:**
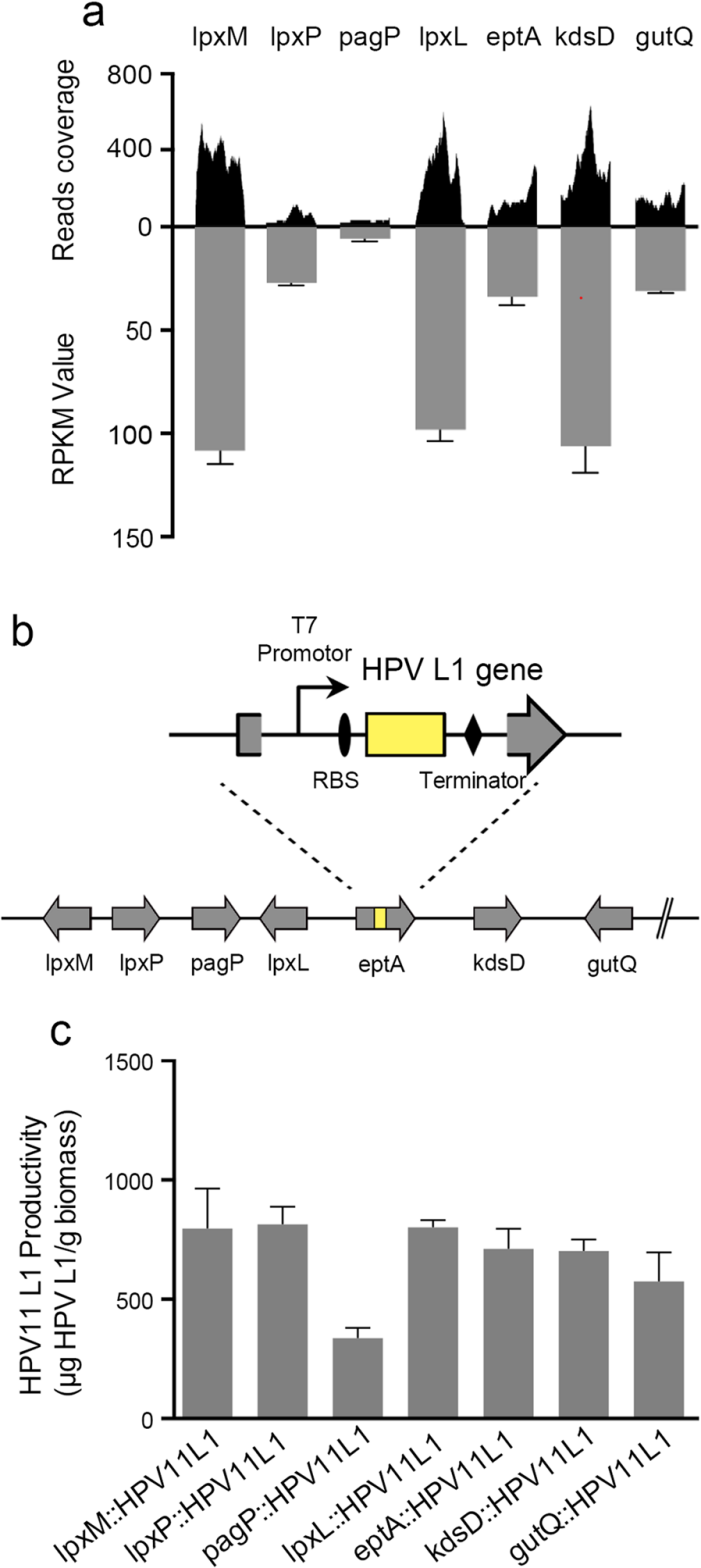
Assessment of LPS synthesis-correlated loci on the chromosome of the *E. coli* ER2566 strain for the integration and expression of the HPV L1 coding sequence. **a** Transcriptome analysis of *E. coli* ER2566 strain was determined via high-throughput sequencing. Reads mapping to the LPS biosynthesis-correlated loci were visualized using Integrative Genomic Viewer (IGV). **b** Schematic map of the HPV L1 protein expression cassette and its integration into the chromosome of the *E. coli* ER2566 strain. This integration interrupted each of the seven loci involved in LPS synthesis. **c** ELISA analysis of the HPV type 11 L1 protein expressed by the constructed *E. coli* strains

According to previous research, chromosomal regions ideal for the insertion of exogenous DNA should be accessible to RNA polymerase and not affect the growth of the recombinant strains. To investigate the potential of these LPS-related loci on the chromosome of the *E. coli* ER2566 strain for integrated expression, we first analyzed the transcriptional activity of these loci during LPS biosynthesis (NCBI accession No. PRJNA598986). After qualifying the sequencing data, high-quality reads were aligned to the *E. coli* ER2566 genome sequence and assessed using the Integrative Genomic Viewer (IGV) [30]. We noticed a remarkable enrichment of reads (≥300) in the open reading frames of *lpxM*, *lpxL*, *eptA*, *kdsD* and *gutQ*, whereas the reads for *pagP* and *lpxP* were less than 200 (Fig. 2a). Using RPKM (Reads Per Kilobase per Million mapped reads) values, we found that, of the 7 loci interrogated, six (*lpxM*, *lpxP*, *lpxL*, *eptA*, *kdsD*, *gutQ*) were more active in transcription (Fig. 2a).

Next, genes encoding for the major capsid protein (L1) of HPV were cloned and integrated into the *E. coli* chromosome as an individual expression cassette under the control of an IPTG-induced T7-promoter (Fig. 2b). Seven recombinant *E. coli* strains carrying a single copy of HPV type 11 (HPV11) L1 protein expression cassette in the LPS synthesis-correlated loci were verified using PCR analysis and DNA sequencing (Additional file 1: Fig. S1a), with the protein expression efficiency measured using an L1-specific antibody, 4B3 (Fig. 2c; Additional file 1: Fig. S2a). Noteworthy, we found that expression was affected by insertion into different regions on the chromosome, with 6 more favorable loci identified: *lpxM*, *lpxP*, *lpxL*, *eptA*, *kdsD* and *gutQ*; the *pagP* locus was deemed unsuitable for HPV11 L1 gene integration, and this result was consistent with the transcriptome sequencing data above.

### Antibiotic-free subcultivation at shake-flask and fermentation scales

To investigate bacterial protein expression during long-term cultivation in the absence of antibiotics, we constructed a recombinant *E. coli* strain, ER2566(*kdsD*::H16) that was genomically integrated with a HPV16 L1 expression cassette and cultured the cells in shake flasks and large-scale fermentation; the parental strain ER2566, harboring an HPV type 16 L1 protein expression plasmid (ER2566(pTO-T7-H16), served as a control.

**Fig. 3:**
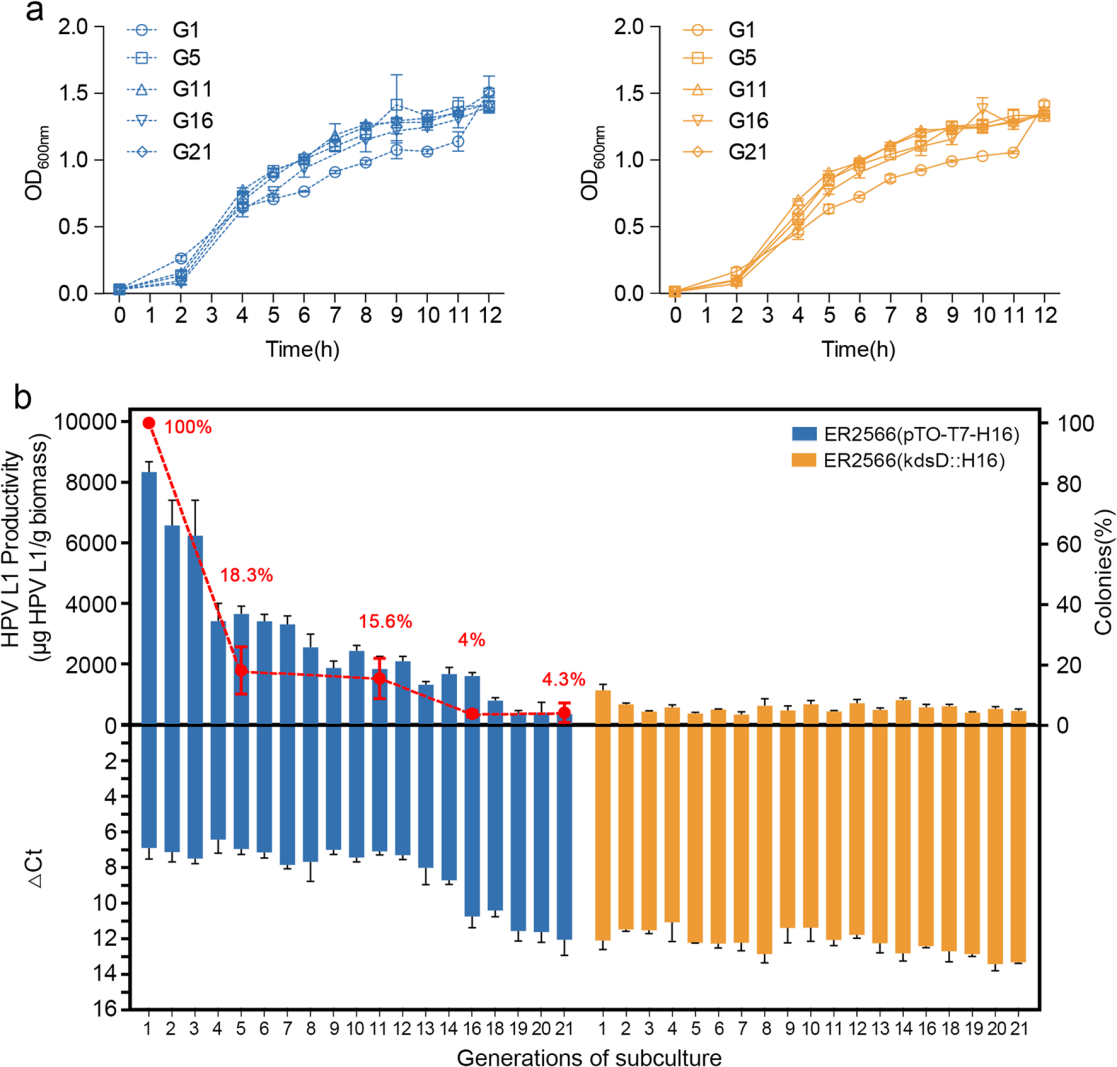
Subcultivation studies of the two *E. coli* strains grown in shake-flasks. **a** Growth curves of the *E. coli* ER2566(pTO-T7-H16) strain (dashed blue lines) and the ER2566(*kdsD*::H16) strain (solid yellow lines) in shake-flask subcultivation. **b** The production of the HPV type 16 L1 protein was determined by ELISA (upper panels). qPCR analysis of the relative transcript levels of the HPV type 16 L1 gene at 6-h post-IPTG induction is shown in the bottom panels as ΔCt values. Plasmid maintenance in the 1st, 5th, 11th, 16th and 21st generations of the ER2566(pTO-T7-H16) strain were determined by measuring the proportion of the strain that contained plasmid (dashed red lines)

In the shake-flask cultivation experiments, the two *E. coli* strains were respectively inoculated into antibiotic-free LB medium, with the OD_600nm_ measured every hour over a 12-h period, referred as one generation. Each generation at the exponential phase (with OD_600nm_ between 0.6 and 1.0) was inoculated into a new flask for subcultivation of the next generation. We successively cultured 21 generations for both *E. coli* strains, and their growth curves, plasmid maintenance, and protein expression levels were evaluated during this period. The *E. coli* strain ER2566(*kdsD*::H16) showed similar growth characteristics to ER2566(pTO-T7-H16); albeit, with slightly different bacterial densities among generations. The OD_600nm_ values increased slowly within the first 2 h and sharply rose to ~1.0 at 5-h post-inoculation. After IPTG induction, the two *E. coli* strains kept growing and reached a plateau phase at an OD_600nm_ 1.2–1.5; the 1st generation (G1) of the two strains had a relatively lower bacterial density than the subcultures throughout the mid- to late-stage of cultivation (Fig. 3a).

Next, we evaluated the HPV L1 protein expression capability of the two strains by measuring transcriptional stability, plasmid maintenance, and protein yield. Cycle threshold (Ct) values were used to determine the transcript yield of the L1 gene in the two strains, with a lower value indicative of higher transcriptional level (negative y-axis). We found an overall higher transcriptional activity of the L1 gene for the ER2566(pTO-T7-H16) strain at the beginning of subcultivation, with ΔCt values between ~6 and ~8 until the 13th generation; this was followed by a steady loss in transcription to ΔCt values of ~12 in subsequent generations (Fig. 3b). This reduction in transcript levels was further confirmed by analyzing plasmid maintenance, in which the proportion of the *E. coli* strain carrying the plasmid sharply dropped to <30% in the 5th generation and on to ~10% by the 16th generation. In the ER2566(*kdsD*::H16) strain, although the transcriptional level of the chromosomally integrated L1 gene was lower than that of the ER2566(pTO-T7-H16) strain, the detected ΔCt values remained relatively stable (between ~12 to ~13), and were comparable with the plasmid-based group in the late subcultivation stage (Fig. 3b).

In regards to the protein expression capability, we found that the ER2566(pTO-T7-H16) strain was more productive than the ER2566(*kdsD*::H16) strain, with an L1 protein productivity of >1500 µg/g in the first 16 generations. However, in the absence of antibiotics, the L1 protein expressed in the plasmid-based group reduced continuously during subcultivation and finally decreased to a comparable level to that of the chromosomally integrated expression group, with expression retained at ~400 to ~600 µg/g. This is consistent with the decline in transcript levels.

**Fig. 4:**
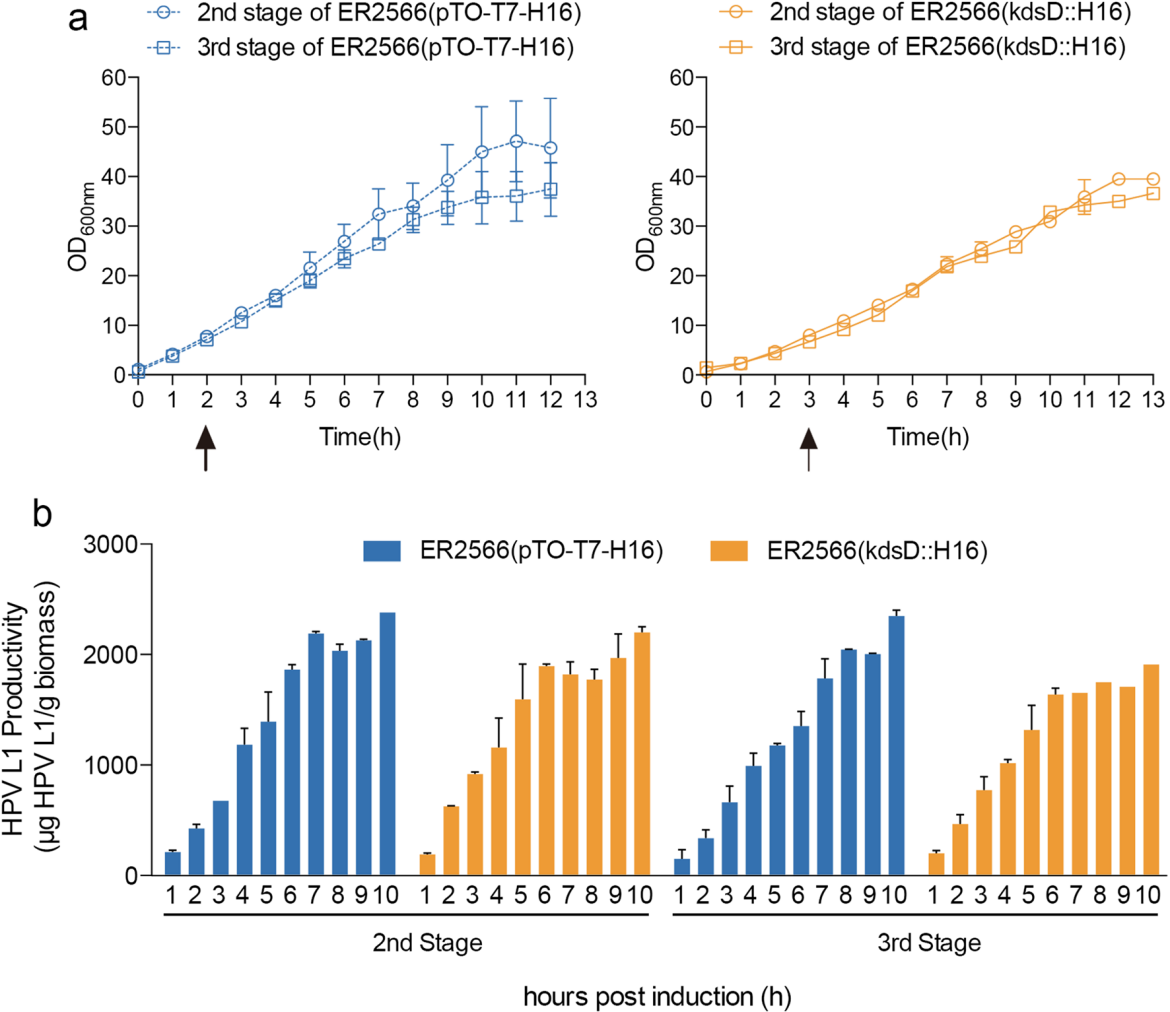
Subcultivation studies of the two *E. coli* strains in large-scale fermentation studies. **a** Growth curves of the *E. coli* ER2566(pTO-T7-H16) strain (dashed blue lines) and the ER2566(*kdsD*::H16) strain (solid yellow lines) during the three-stage fermentation process. The time points for IPTG induction are indicated by the black arrows. **b** HPV type 16 L1 protein production during fermentation was analyzed by ELISA

Continuous high-density fermentation is often used to increase biomass and protein productivity of various bacteria and virus strains. Here, the *E. coli* strains, ER2566(*kdsD*::H16) and ER2566(pTO-T7-H16), were used for fermentation, which was carried out in three 5-l fermenters to simulate a three-stage fermentation process. The two strains were respectively inoculated in antibiotic-free OB16 medium and cultivated at 37 °C. Cultures at an OD_600nm_ of ~4.0 were partially seeded to the next stage of fermentation and chilled to 20 °C for L1 protein expression. Growth curves, plasmid maintenance, and cell productivity toward L1 protein production were evaluated in the 2nd and 3rd stages of fermentation. We found that bacterial density during fermentation persistently increased until reaching a plateau phase (Fig. 4a). The growth rate of the ER2566(*kdsD*::H16) strain was slower than that of the ER2566(pTO-T7-H16) strain with about a 1-h delay. Whereas the growth curves in the ER2566(pTO-T7-H16) strain varied from batch to batch, the ER2566(*kdsD*::H16) strain was stable. Production of the L1 protein was monitored once every hour after IPTG induction, and was shown to increase gradually along with the growth of the two strains. Notably, the ER2566(*kdsD*::H16) strain produced a comparable amount of L1 protein to that of the ER2566(pTO-T7-H16) strain, with values between 2000 µg/g and 2500 µg/g (Fig. 4b). The reduced productivity of the ER2566(pTO-T7-H16) strain at the fermentation scale may result from a loss of plasmid during continuous cultivation in the absence of antibiotics (Additional file 1: Fig. S3).

Overall, we show that the two *E. coli* strains grown via different expression strategies (plasmid-based or bacterial chromosome-based) showed similar growth properties yet different levels of L1 protein production. Therefore, with the advantages of stable productivity and antibiotic-free cultivation, *E. coli* strains containing chromosomally integrated expression cassettes may provide an alternative way of scaling up the production of recombinant proteins.

### Construction and evaluation of *E. coli* strains containing multiple copies of the chromosomally integrated HPV L1 expression cassette

**Fig. 5:**
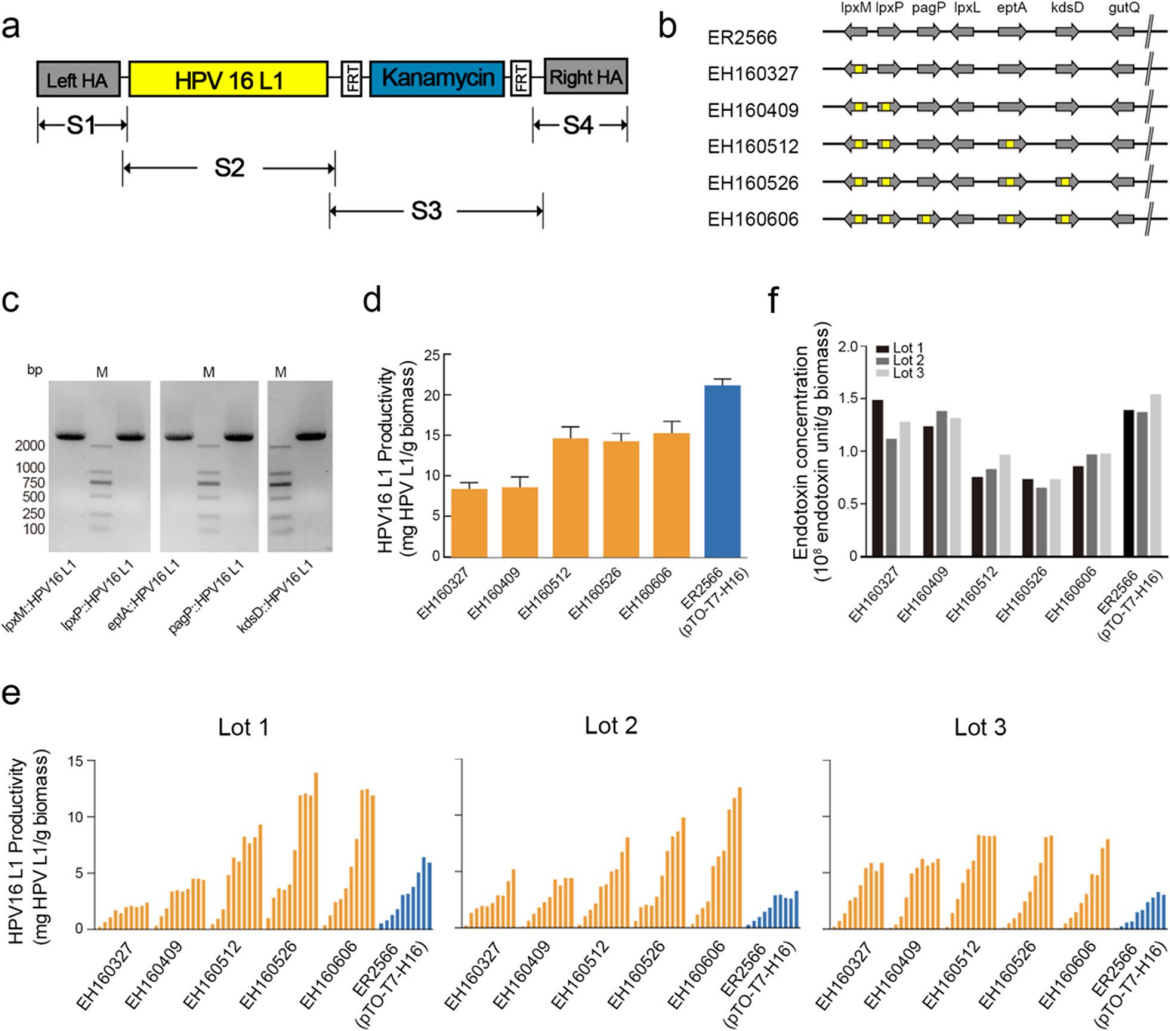
Construction and evaluation of *E. coli* strains containing multi-copies of the chromosomally integrated expression cassette. **a** Schematic map of the donor DNA. The S1, S2, S3 and S4 fragments of the donor DNA were respectively amplified and joined together using overlap extension PCR. **b** Schematic diagram of the different recombinant strains bearing 2-5 copies of the integrated expression cassettes. **c** PCR analysis of the resultant *E. coli* strains at each gene editing step. Lanes marked with “M” indicate the DNA marker. **d** Production of the HPV type 16 L1 protein prepared using shake-flasks was determined by ELISA. **e** The expression capacity of the HPV type 16 L1 protein in the *E. coli* strains was evaluated in 3 fermentation batches in the absence of antibiotics, and measured by ELISA. **f** The endotoxin level of the bacterial lysates was detected by TAL assay

The copy number of the expression cassette is an important factor influencing protein expression. To improve the production of HPV L1 protein in *E. coli* using the bacterial chromosome-based expression strategy, we constructed five *E. coli* strains bearing 1 to 5 copies of the expression cassettes, respectively. The donor DNA (Fig. 5a) was amplified and integrated into the *lpxM*, *lpxP*, *pagP*, *eptA*, and *kdsD* loci of the ER2566 strain separately through lambda-Red recombination (Fig. 5b and c). The constructed *E. coli* strains are shown in Table 2.

**Table 2:** Summary of the constructed *E. coli* strains

Strains	Relevant genotype	Source or reference
ER2566	F- λ- fhuA2 [lon] ompT lacZ::T7 gene 1 gal sulA11 Δ(mcrC-mrr)114::IS10 R(mcr-73::miniTn10-TetS)2 R(zgb-210::Tn10)(TetS) endA1 [dcm]	Laboratory strain
ER2566(pTO-T7-H16)	ER2566 harboring pTO-T7-HPV16L1 plasmid	This study
ER2566(lpxL::H11)	ER2566(lpxL::HPV11 L1)	This study
ER2566(lpxM::H11)	ER2566(lpxM::HPV11 L1)	This study
ER2566(lpxP::H11)	ER2566(lpxP::HPV11 L1)	This study
ER2566(eptA::H11)	ER2566(eptA::HPV11 L1)	This study
ER2566(pagP::H11)	ER2566(pagP::HPV11 L1)	This study
ER2566(kdsD::H11)	ER2566(kdsD::HPV11 L1)	This study
ER2566(gutQ::H11)	ER2566(gutQ::HPV11 L1)	This study
ER2566(lpxL::H18)	ER2566(lpxL::HPV18 L1)	This study
ER2566(lpxM::H18)	ER2566(lpxM::HPV18 L1)	This study
ER2566(lpxP::H18)	ER2566(lpxP::HPV18 L1)	This study
ER2566(eptA::H18)	ER2566(eptA::HPV18 L1)	This study
ER2566(pagP::H18)	ER2566(pagP::HPV18 L1)	This study
ER2566(kdsD::H18)	ER2566(kdsD::HPV18 L1)	This study
ER2566(gutQ::H18)	ER2566(gutQ::HPV18 L1)	This study
ER2566(lpxL::H52)	ER2566(lpxL::HPV52 L1)	This study
ER2566(lpxM::H52)	ER2566(lpxM::HPV52 L1)	This study
ER2566(lpxP::H52)	ER2566(lpxP::HPV52 L1)	This study
ER2566(eptA::H52)	ER2566(eptA::HPV52 L1)	This study
ER2566(pagP::H52)	ER2566(pagP::HPV52 L1)	This study
ER2566(kdsD::H52)	ER2566(kdsD::HPV52 L1)	This study
ER2566(gutQ::H52)	ER2566(gutQ::HPV52 L1)	This study
ER2566(kdsD::H6)	ER2566(kdsD::HPV6 L1)	This study
ER2566(kdsD::H16)	ER2566(kdsD::HPV16 L1)	This study
ER2566(kdsD::H31)	ER2566(kdsD::HPV31 L1)	This study
ER2566(kdsD::H33)	ER2566(kdsD::HPV33 L1)	This study
ER2566(kdsD::H45)	ER2566(kdsD::HPV45 L1)	This study
ER2566(kdsD::H52)	ER2566(kdsD::HPV52 L1)	This study
ER2566(kdsD::H58)	ER2566(kdsD::HPV58 L1)	This study
EH160327	ER2566(lpxM::HPV16 L1)	This study
EH160409	EH160327(lpxP::HPV16 L1)	This study
EH160512	EH160409(eptA::HPV16 L1)	This study
EH160526	EH160512(kdsD::HPV16 L1)	This study
EH160606	EH160526(pagP::HPV16 L1)	This study

The ER2566(pTO-T7-H16) strain and five recombinant *E. coli* strains with increasing copy numbers of the expression cassettes were cultivated in shake-flasks. L1 protein levels were determined by ELISA and western blotting (Fig. 5d; Additional file 1: Fig. S4). Consistent with the production in the ER2566(*kdsD*::H16) strain, the productivity in the cultures of the EH160327 strain were about half to one-quarter of that in cultures from the parental ER2566(pTO-T7-H16) strain. However, among the recombinant strains, higher levels of the L1 protein were produced with higher copy numbers of the integrated expression cassette. Indeed, the EH160512, EH160526 and EH160606 strains with 3 to 5 copies of expression cassette, respectively, produced a comparable level of HPV16 L1 protein as that of the ER2566(pTO-T7-H16) strain (~1.5 × 10^4^ µg/g vs. ~2.0 × 10^4^ µg/g, Fig. 5d).

We next evaluated HPV 16 L1 protein production from recombinant strains in a scale-up fermentation in a 5-liter bioreactor containing OB16 medium. Three batches of the L1 protein for each *E. coli* strain were produced and monitored once per hour for 10 h. The relative transcript level of the L1 gene was analyzed by qPCR at 0 h, 1 h, and 6 h post-IPTG addition. Similar growth curves were observed for the cultivated *E. coli* strains; albeit, bacterial density in the final cultures differed among batches. The OD_600nm_ values increased sharply over the first 8 h and slowly rose and plateaued at ~25 to ~40 (Additional file 1: Fig. S5a). Not surprisingly, we obtained a higher quantity of L1 proteins in the large-scale fermentation as compared with that achieved from the shake-flask culture, with a similar increase in yield and a plateau phase of production as growth slowed down.

We next ascertained and compared the expression cassette copy number in these cultures. The recombinant *E. coli* strains EH160327 and EH160409, bearing single and double copies of the expression cassette, produced comparable levels of L1 protein (~2.4 mg/g vs. ~4.4 mg/g for lot 1; ~5.1 mg/g vs. ~4.4 mg/g for lot 2; and ~5.8 mg/g vs. ~6.2 mg/g for lot 3; Fig. 5e). When the *E. coli* strains were chromosomally integrated with 3 to 5 copies of the expression cassette, the L1 protein production was further enhanced by 1.5- to 2-fold, with between ~8.0 mg/g to ~14 mg/g detected (Fig. 5e). As expected, the parental strain, ER2566(pTO-T7-H16), produced less L1 protein during high-density cultivation without antibiotic supplementation, which was comparable with that produced by the recombinant strains with 1- or 2-copies of the expression cassette. The differences in production amounts were further demonstrated by the relative transcript levels of the L1 gene (Fig. S5b). Finally, we tested the bacteria from the fermentation batches for endotoxin levels using the TAL assay. The endotoxin EU equivalents decreased with increasing copies of the expression cassettes which were integrated in the genes correlated with LPS biosynthesis. The parental strain ER2566 harboring the expression plasmid, however, was similar to that of the recombinant strains that contained 1 or 2 copies of the expression cassette, and this similarity was consistent across all three test batches (1.1 × 10^8^ EU/g to 1.5 × 10^8^ EU/g; Fig. 5f). Recombinant strains containing 3 to 5 copies of the expression cassette had a 1.5- to 2-fold lower endotoxin level than the parental strain. Taken together, integrating 3- to 5-copies of the expression cassette for the exogenous gene into a bacterial LPS-correlated loci not only enhances the production of the target protein but also reduces the production of the prokaryote-derived endotoxin.

### Construction and application of recombinant *E. coli* strains for the production of capsid proteins of an HPV 9-valent vaccine candidate

To further characterize the chromosomally integrated expression of other genotypes of the HPV L1 protein, we constructed another 8 recombinant *E. coli* strains in addition to that for HPV 11 L1 with the aim of creating an HPV 9-valent vaccine. An HPV 9-valent vaccine has been developed previously with well-characterized VLPs using a plasmid-based *E. coli* expression system [31] and has been tested in phase 3 clinical trials in China (Register no. 2017L04931) offering benefit over lower valency vaccines against HPV.

**Fig. 6:**
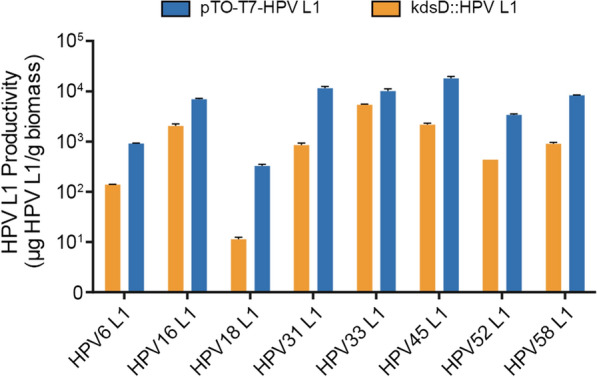
ELISA analysis of the L1 proteins of the HPV types 6, 16, 18, 31, 33, 45, 52 and 58 expressed by the *E. coli* strains via chromosomal integration (*kdsD*::HPV L1; orange) or plasmid-based expression strategy (pTO-T7-HPV L1; blue)

Here, we first introduced each expression cassette into the *kdsD* site for the L1 genes of HPV types 6, 16, 18, 31, 33, 45, 52 and 58 (Additional file 1: Fig. S1b) via integration into the bacterial chromosome. We then analyzed the expression of the produced HPV L1 proteins using ELISA and western blotting (Fig. 6; Additional file 1: Fig. S2b). We found a higher productivity of the *E. coli* cells via the plasmid-based strategy, with the expression pattern differing among the genotypes. Indeed, whereas the amount of the HPV type 33 L1 protein expressed by the chromosomally integrated cassette was close to that produced via plasmid-based incorporation (~5400 µg/g vs. ~10,200 µg/g), for the rest HPV genotypes (6, 16, 18, 31, 45, 52 and 58), the L1 protein produced by the genetically integrated strains ranged from ~11 µg/g to ~2200 µg/g, measured to be 3- to 28-times lower than that of the parental ER2566 strain with the corresponding expression plasmid. The chromosomally integrated strains for HPV type 6, 18 and 52 presented with the lowest levels of L1 protein production, with less than 500 µg/g in the ELISA analysis, and weak detection in western blotting (particularly HPV type 18 and 52 L1 protein). This low expression capability of the genetically integrated strains may have resulted from the expression cassette copy number and the influence of its insertion into the *kdsD* locus.

To rectify this, we evaluated the potential of other loci for the integrated expression of HPV type 18 and 52 L1 genes (Additional file 1: Fig. S1c,  S1d). We found that when the expression cassettes were integrated into *lpxM*, *pagP* and *gutQ* loci, the L1 protein expression level increased (Additional file 1: Fig. S2c, S2d), suggesting preferential chromosomal locations for the insertion of these two genotypes of the HPV L1 gene.

**Fig. 7:**
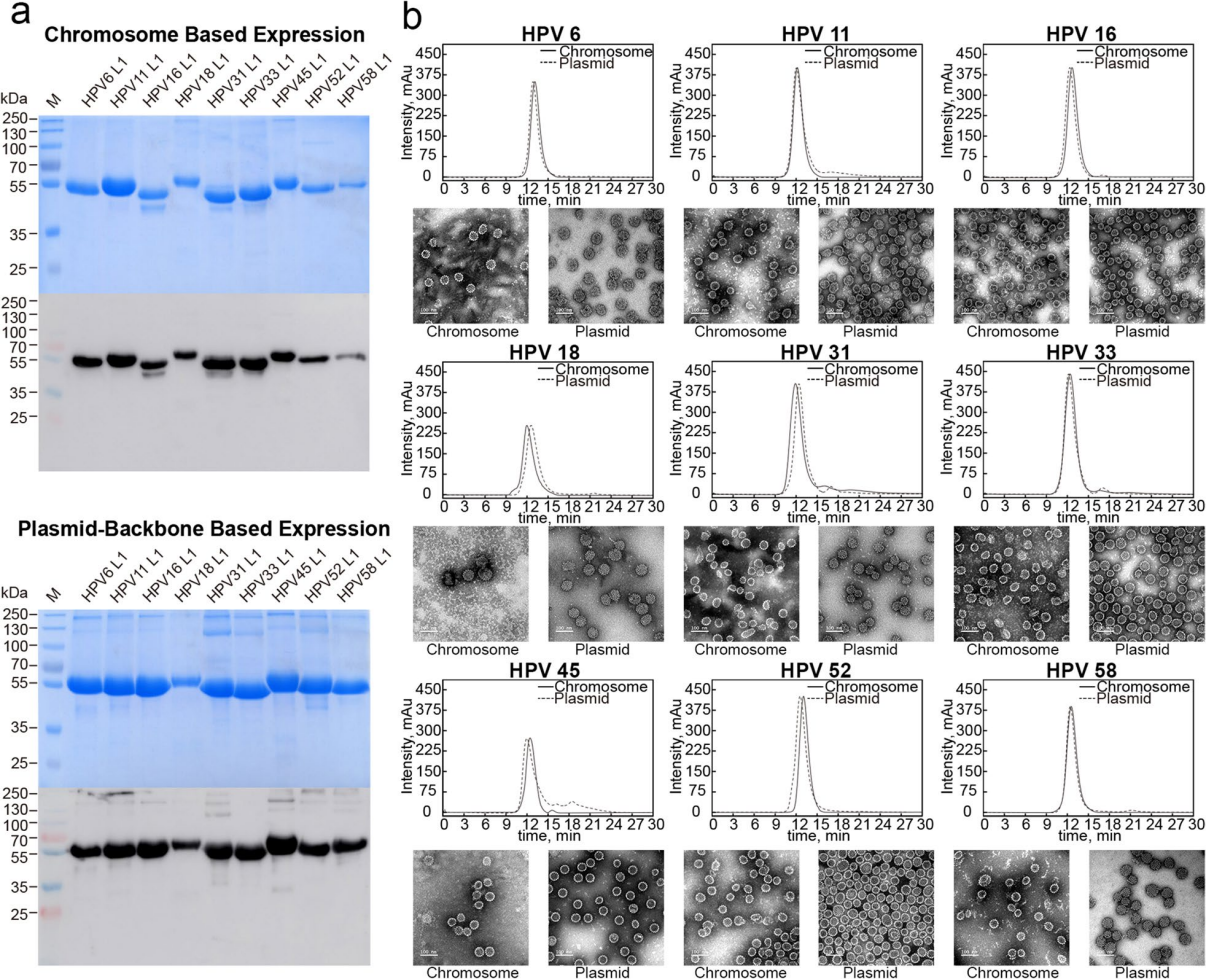
Evaluation of the HPV 9-valent capsid proteins produced via two expression strategies in *E. coli* strains. **a** The purified 9 genotypes of the HPV L1 protein were analyzed by SDS-PAGE and western blotting. The broad cross-genotype mAb 4B3 was used for detection in western blotting. The lanes marked with an “M” indicate the protein marker. **b** The morphologies of the HPV L1 particles were visualized by transmission electron microscopy, and the molecular sizes of HPV L1 particles were evaluated by HPSEC

All of the *E. coli* strains were cultured in antibiotic-free LB medium and induced by IPTG. The L1 proteins were purified to considerably high purity and expression confirmed by SDS-PAGE and western blotting (Fig. 7a). All of the HPV 9-valent L1 proteins, expressed either by chromosomal integrated expression cassette or plasmid, reacted well with the HPV L1-specific antibody 4B3. Additionally, negative-stain transmission electron microscopy confirmed that all of the 9-valent L1 proteins expressed from the *E. coli* chromosomal cassettes were able to self-assemble into virus-like particles (VLPs) with an average diameter of 50 nm, with a similar morphology noted for the VLPs achieved by plasmid expression (Fig. 7b). Finally, high-performance size-exclusion chromatography (HPSEC) confirmed a similar molecular weight for VLPs achieved by the two expression strategies in terms of relative retention time (Fig. 7b).

Taken together, the HPV L1 VLPs derived from genome constitutive expression share comparable properties with those from plasmid-based expression, suggesting that this strategy is amenable to vaccine and recombinant protein development in bioindustry.

## Discussion

*E. coli* is one of the most widely used cell factories for the expression of recombinant proteins and is a standard practice in the biopharmaceutical field. Due to the characteristics of Gram-negative bacteria and their exogenous expression vectors, therapeutic drugs derived from prokaryotes need to be strictly assessed before they can be certified for human use, and tested for the residual presence of endotoxins and antibiotics. Previous studies have demonstrated that a detoxified *E. coli* strain could be used to produce recombinant proteins as inclusion bodies or soluble states. Compared with recombinant proteins in wild-type *E. coli* strains, recombinant proteins manufactured by LPS-deficient *E. coli* strains rarely stimulate an inflammatory reaction—particularly from the intracellular TLR4/MD-2 signaling pathway—leading to enhanced safety. Furthermore, plasmid-mediated expression of recombinant proteins in *E. coli* in large-scale multiple fed-batch cultivation in the absence of antibiotics is unstable. The integration of the target gene or even an expression cassette into the bacterial chromosome is thus an alternate appealing strategy for producing recombinant proteins or chemicals.

Thus, herein, we attempted to construct an *E. coli* strain that could stably express recombinant proteins while generating low intrinsic levels of endotoxin. We disrupted 7 LPS synthesis-associated loci through integration of the expression cassettes of HPV L1 genes, and evaluated whether the resulting *E. coli* strains were more suitable for the production of recombinant proteins in high-scale fermentation. We found that the *E. coli* strains with chromosomally integrated expression cassettes retained a similar growth rate as the parental cells and a more stable protein-expression capability than plasmid-based expression. As to protein production, a similar level of HPV L1 protein was produced in the final large-scale fermentation products of the two *E. coli* strains, and this could be significantly enhanced (1.5- to 2-fold) with a higher copy number of chromosomally integrated expression cassettes. Moreover, the functional and structural features of the HPV L1 VLPs manufactured using the constructed *E. coli* strains were also comparable with that of the parental *E. coli* strains. Although we have proved the availability for overexpression of the HPV 9-valent vaccine candidate from bacterial chromosome, multiple expression cassettes should be tried to integrate into more LPS synthesis-associated loci for lower endotoxin residual and higher expression yield, which is indeed valuable in vaccine industrial practice.

Additionally, the exact position wherein to incorporate the exogenous genes for expression remains to be studied. Earlier work showed that the output of a foreign expression cassette is influenced by the integrated location on the bacterial chromosome, as well as the culture conditions, exposure to environmental stress, and metabolic burden. We thus assessed seven LPS synthesis-associated loci, including *lpxL*, *lpxM*, *lpxP*, *eptA*, *pagP*, *kdsD* and *gutQ*, as locations for the insertion and expression of HPV L1 proteins. We found that the transcriptional activity of the endogenous genes and the nucleotide component of the integrated exogenous genes affect the production capacity of the cells. The three loci with the best RNA polymerase accessibility, *lpxL*, *lpxM* and *kdsD*, may serve as good locations for the integrated expression of HPV L1 proteins.

Taken together, chromosomal integration of the expression cassettes on the *E. coli* LPS-correlated loci largely reduces the residual endotoxin in the final products and makes it unnecessary to supply antibiotics for plasmid maintenance during continuous, high-density fermentation. Although the manipulation of chromosomal integration is more complicated than plasmid transformation to bacterium, the most beneficial of the former in protein expression is plasmid-free steady and antibiotic-free culture while multiple continuous batches of fermentation in large-scale industrial production. The constructed *E. coli* strains may provide a new approach for the production of recombinant protein vaccines or drugs at an industry scale.

## Conclusions

In this study, we successfully constructed 7 LPS-defective synthetic strains and significantly reduced residual endotoxin levels in the protein products as compared with the wild-type strain. We evaluated the loci associated with LPS synthesis in terms of transcription efficacy in response to the integrated expression of exogenous genes. Further, we constructed and engineered 9 recombinant *E. coli* strains for the later development of an HPV 9-valent capsid with desirable purity, VLP morphology and antigenicity. These results may provide a new strategy for recombinant *E. coli* strain construction and the development of other recombinant vaccines.

## Methods

### Strains, plasmids and culture conditions

The *E. coli* strain ER2566 (New England Biolabs; Beverly, MA, USA) was used as the parental strain for characterizing chromosomal integrated expression. Plasmids pKD46 (CGSC #7739), pkD4 (CGSC #7632) and pCP20 (CGSC #7629) were purchased from Coli Genetic Stock Center (CGSC, Yale University; New Haven, CT, USA). Plasmids pCas (Addgene #62225) and pTargetF (Addgene #62226) were gifts from Dr. Jiang Yu (Shanghai Institutes for Biological Sciences). Plasmids for expressing the L1 proteins of various HPV types (6, 11, 16, 18, 31, 33, 45, 52, 58) were previously constructed and described elsewhere [31].

LB medium, OB16 medium, and SOC medium were used for shake-flask cultivation, high-density continuous fermentation, and cell recovery, respectively. Kanamycin and spectinomycin were used at final concentrations of 25 µg/ml and 50 µg/ml, respectively. l-arabinose was added to a final concentration of 10 mM to induce the expression of RED recombinase during the preparation of competent cells. IPTG at a final concentration of 0.8 mM was used to induce the expression of HPV L1 proteins and pTargetF plasmids.

### Genes editing of the parental *E. coli* strain ER2566

Bacterial chromosome modification (target gene deletion, single nucleotide mutation and exogenous genes insertion) was accomplished via homologous recombination or homology-directed repair (HDR).

Primers were designed to construct gRNA plasmids and donor DNA via PCR amplification. Spacer sequences targeting each genomic locus were amplified as short dsDNA and then inserted into pTargetF plasmid via Gibson Assembly. To construct donor DNA, an exogenous gene expression cassette and two homology arms were separately amplified and then joined together by overlap extension PCR. DpnI enzyme was added to the final PCR product to digest the plasmid template.

Electrocompetent cells were optimized for genome editing. The *E. coli* strain ER2566 containing the helper plasmid (pKD46 or pCas plasmid) was inoculated in LB medium with appropriate antibiotics, and incubated at 30 °C to an OD_600_ of 0.6. l-arabinose was added to the cultures to induce Red recombinase. Cells were then quickly chilled on ice for 30 min and then washed thrice using cold, sterile double-distilled water and stored in 10% v/v glycerol. Competent cells were transformed with 300 ng donor DNA, or with 300 ng donor DNA and 200 ng pTarget plasmid, respectively. After electroporation, the cells were immediately added into SOC medium, cultivated for 1 h at 37 °C, and then spread onto LB plates containing the appropriate antibiotics. Transformants were screened using PCR amplification and sequencing for correct genomic modifications.

### HPV L1 protein expression, purification and VLP assembly

The recombinant strain carrying the HPV L1 protein expression cassette was inoculated into 15 flasks containing LB medium without antibiotic for a total volume of 7.5 l. When cells reached the exponential phase, the cultivation temperature was shifted from 37 °C to 20 °C, and IPTG was added into cultures to initiate transcription of the HPV L1 expression cassette located on the ER2566 chromosome. After 12 h, cells were harvested and disrupted by sonication. The HPV L1 protein was purified and assembled in the form of VLPs, according to a previous research [31].

### Antigenicity and morphology of VLP

VLPs from engineered strains were characterized and compared with those from the parental strain. Purified HPV L1 proteins were mixed with loading buffer and heated at 80 °C for 10 min. The sample mixtures were then loaded into the wells of 10% acrylamide gels and subjected to electrophoresis. For western blotting analysis, gels were transferred onto nitrocellulose membranes. Membranes were immersed in blocking solution (5% non-fat milk in phosphate-buffered saline (PBS), pH 7.45) for 1 h, and then incubated with monoclonal antibody 4B3, which recognizes a broad and linear epitope of different types of HPV L1 proteins. After three washes with PBST, the membranes were incubated with HRP-conjugated secondary antibody to detect the bound primary antibody. The interaction signal was examined using ImageQuant LAS4000mini.

The HPV L1 VLPs were prepared at a concentration of 0.5 mg/ml and absorbed onto carbon-coated grids for staining with 2% sodium phosphotungstate (pH = 7.0). VLP morphology was visualized using a Tecnai G2 Spirit transmission electron microscope (FEI, Oregon, USA).

### Analysis of plasmid stability

Samples from fermentation broth were diluted to appropriate concentrations and spread onto antibiotic-free LB plates. After incubation at 37 °C for 14–17 h, 100 single colonies were randomly picked, and each colony transferred to LB plates with antibiotics and plates without antibiotics. Plasmid stability was determined by the percentage of colonies formed on the selective LB plates after 14–17 h cultivation at 37 °C. Two parallel of selective and non-selective LB plates were utilized to calculate the mean percentage of plasmid-bearing cells.

### Real-time PCR

Real-time PCR was conducted to determine HPV16 L1 gene expression patterns. Total RNA was isolated using the RNAprep Pure cell/Bacteria kit (TIANGEN, Beijing). One-step qPCR was performed using the listed primers (16sRNA F: 5′-TGATAAACTGGAGGAAGGTG-3′ R: 5′-CACTTTATGAGGTCCGCTTG-3′, HPV16L1 F: 5′- GTCCCAGTATCTAAGGTTGT-3′, R 5′-GGTTTTTTAATAGGAAAATA-3′) and probes (16sRNA, 5′- TGGCCCTTACGACCAGGGCT-3′, HPV16L1, 5′-TCATGCAGGAACATCCAGAC-3′) with the following reaction volumes: 1 µl template, 0.5 µl F/R primers, 64 µl of 10× buffer, 2 µl of 2.5 mM dNTP, 0.4 µl HS Taq, 0.2 µl TransScript II Reverse Transcriptase (TransGen Biotech, Beijing), 10.4 µl DEPC H_2_O, and 1 µl probe. The PCR program was run at 50 °C for 10 min, 95 °C for 10 min, followed by 45 cycles of 95 °C for 15 s and 55 °C for 50 s. Relative mRNA levels were evaluated using the comparative CT (2^−△Ct^) method, with 16 S RNA used as the internal reference.

### Endotoxin measurement

The concentration of LPS in the supernatants of cell lysates was determined by the kinetic turbidimetric assay using the tachypleus amebocyte lysate (TAL) test reagent kit purchased from Xiamen Bioendo Technology Co., Ltd. The supernatant was diluted with endotoxin-free water. The lyophilized endotoxin standard stock was reconstituted and diluted to final concentrations of 10, 1, 0.1, 0.05, and 0.01 EU ml^− 1^. All samples, standards and negative controls were placed into the wells of a 96-well plate (non-pyrogens) in duplicate. 100 µl TAL reagent was added into each well, and the plate was incubated at 37 °C in a BioTek ELx808 reader (BioTek Instruments, Inc.) for 2 h. The endotoxin level was measured using a kinetic assay program and calculated using Gen5™ data analysis software (BioTek Instruments, Inc.).

### Quantification of HPV L1 protein in supernatant of cell lysis

To determine the level of HPV L1 protein expression in fermentation scale, recombinant cells and parental cells were harvested and disrupted by sonication. HPV L1 protein in the supernatant of cell lysates was determined by mAb-based sandwich ELISA analysis. The 96-well microplates were coated with HPV L1 type-specific mAbs at suitable concentrations to capture HPV L1 protein in serially diluted supernatant samples. An HRP-conjugated type-specific mAb was used as detection antibody.

## Supplementary Information

**Additional file 1:** **Fig. S1**. The PCR analysis of the reconstructed *E. coli* strains. (a) PCR analysis of the constructed *E. coli* strains with a single copy of the HPV type 11 L1 gene expression cassette integrated at the  *lpxM*, *lpxP*, *pagP*, *lpxL*, *eptA*, *kdsD* or *gutQ* locus of the parental *E. coli *ER2566 strain. (b) Expression cassettes were constructed for the production ofthe L1 protein from 8 HPV genotypes, and were separately integrated in the* kdsD* locus of the *E. coli* ER2566 strain. (c) and (d) The *lpxL*, *lpxM*, *lpxP*, *eptA*, *pagP* and *gutQ* loci were evaluated for the integrated expression of HPV type 18 and 52 L1 genes. The reconstructed *E. coli* strains were analyzed by PCR. **Fig. S2**. SDS-PAGE and western blotting analysis of the HPV L1 protein (produced at the shake-flask level). Lanes marked with “M” indicate the protein marker. Lanes marked with “+” indicate the HPV type 16 L1 protein with high purity, which was used as a positive control. Lanes marked with “P” indicate the HPV L1 protein expressed by the *E. coli *strain utilizing a plasmid-based strategy. Lanes marked with “C_gene locus_” indicate the HPV L1 protein expressed by the *E. coli* strain utilizing chromosomally integrated expression strategy. (a) The HPV type 11 L1 protein was, respectively, expressed from the cassette integrated into 7 loci of the ER2566 strain. (b) L1 protein from HPV types 6, 16, 18, 31, 33, 45, 52, and 58 were, respectively, expressed from the cassette integrated into the *kdsD* locus of the ER2566 strain. (c) and (d) L1 protein from HPV types 18 and 52 were, respectively, expressed from the cassette integrated into 6 loci (as indicated above) of the ER2566 strain. **Fig. S3**. Evaluation of plasmid maintenance during the three-stage fermentation process. **Fig. S4**. SDS-PAGE and western blotting analysis of the HPV type 16 L1 protein expressed by the constructed *E. coli *strains bearing multiple copies of the integrated expression cassette. Lanes marked with “M” indicate the protein marker. Lanes marked with “+” indicate the HPV type 16 L1 protein with high purity, which was used as a positive control. **Fig. S5**. Growth curves (a) and transcript levels (b) of the recombinant *E. coli* strains bearing multiple copies of the HPV16 target gene during large-scale fermentation. The time points were set as 0, 1, and 6 h post-IPTG induction.

## Data Availability

The RNA-Seq raw data used in this study had been deposited in NIH Sequence Read Archive under accession number: PRJNA598986.

## Abbreviations

*E. coli*: *Escherichia coli*
LPS: Lipopolysaccharide
IGV: Integrative genomic viewer
RPKM: Reads per kilobase per million mapped reads
TAL: Tachypleus amebocyte lysate assay
EU: Endotoxin unit
HEV: Hepatitis E virus
HPV: Human papillomavirus
IPTG: Isopropyl-beta-d-thiogalactopyranoside
