# Supplementary material for: Engineering for an HPV 9-valent vaccine candidate using genomic constitutive over-expression and low lipopolysaccharide levels in Escherichia coli cells
Source: Microb Cell Fact. 2021 Dec 20;20:227. doi: 10.1186/s12934-021-01719-8 (PMC8686089; doi:10.1186/s12934-021-01719-8)
Supplement: Supplementary file 1 — Additional file 1: Fig. S1. The PCR analysis of the reconstructed E. coli strains. (a) PCR analysis of the constructed E. coli strains with a single copy of the HPV type 11 L1 gene expression cassette integrated at the lpxM, lpxP, pagP, lpxL, eptA, kdsD or gutQ locus of the parental E. coli ER2566 strain. (b) Expression cassettes were constructed for the production ofthe L1 protein from 8 HPV genotypes, and were separately integrated in the kdsD locus of the E. coli ER2566 strain. (c) and (d) The lpxL, lpxM, lpxP, eptA, pagP and gutQ loci were evaluated for the integrated expression of HPV type 18 and 52 L1 genes. The reconstructed E. coli strains were analyzed by PCR. Fig. S2. SDS-PAGE and western blotting analysis of the HPV L1 protein (produced at the shake-flask level). Lanes marked with “M” indicate the protein marker. Lanes marked with “+” indicate the HPV type 16 L1 protein with high purity, which was used as a positive control. Lanes marked with “P” indicate the HPV L1 protein expressed by the E. coli strain utilizing a plasmid-based strategy. Lanes marked with “Cgene locus” indicate the HPV L1 protein expressed by the E. coli strain utilizing chromosomally integrated expression strategy. (a) The HPV type 11 L1 protein was, respectively, expressed from the cassette integrated into 7 loci of the ER2566 strain. (b) L1 protein from HPV types 6, 16, 18, 31, 33, 45, 52, and 58 were, respectively, expressed from the cassette integrated into the kdsD locus of the ER2566 strain. (c) and (d) L1 protein from HPV types 18 and 52 were, respectively, expressed from the cassette integrated into 6 loci (as indicated above) of the ER2566 strain. Fig. S3. Evaluation of plasmid maintenance during the three-stage fermentation process. Fig. S4. SDS-PAGE and western blotting analysis of the HPV type 16 L1 protein expressed by the constructed E. coli strains bearing multiple copies of the integrated expression cassette. Lanes marked with “M” indicate the protein marker. Lanes mar [file 12934_2021_1719_MOESM1_ESM.docx]

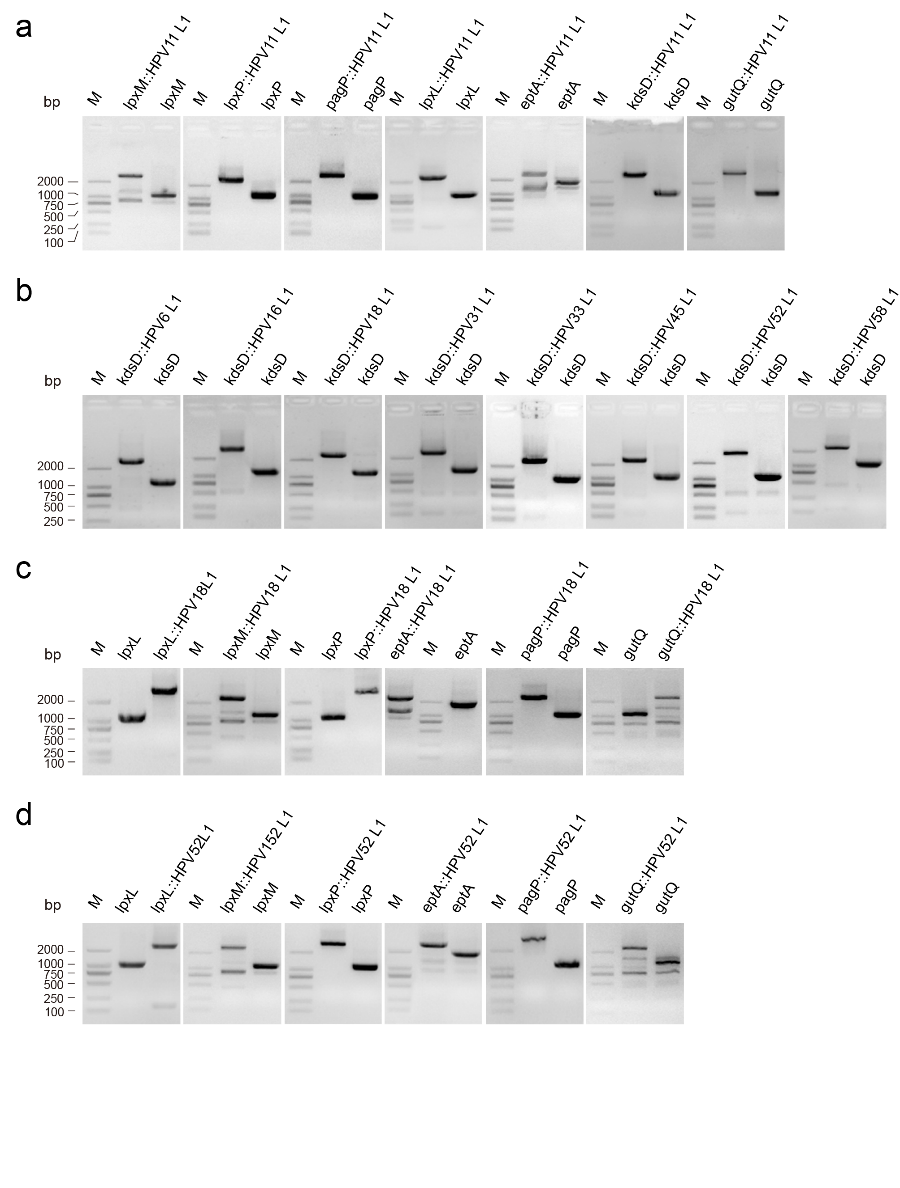


Fig. S1. (a) PCR analysis of the constructed *E. coli* strains with a single copy of the HPV type 11 L1 gene expression cassette integrated at the lpxM, lpxP, pagP, lpxL, eptA, kdsD or gutQ locus of the parental *E. coli* ER2566 strain. (b) Expression cassettes were constructed for the production of the L1 protein from 8 HPV genotypes, and were separately integrated in the kdsD locus of the *E. coli* ER2566 strain. (c) and (d) The lpxL, lpxM, lpxP, eptA, pagP and gutQ loci were evaluated for the integrated expression of HPV type 18 and 52 L1 genes. The reconstructed *E. coli* strains were analyzed by PCR.


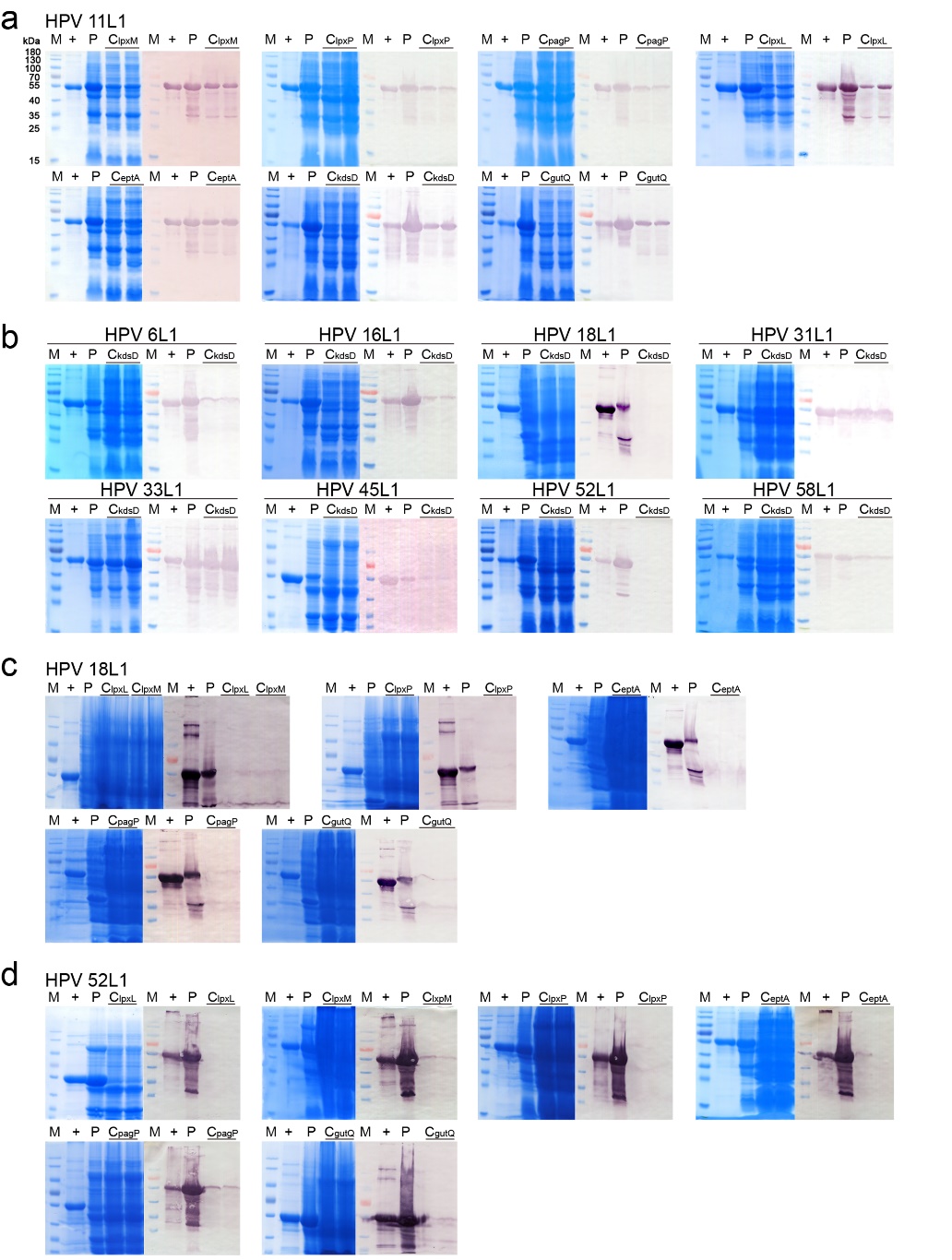


Fig. S2. SDS-PAGE and western blotting analysis of the HPV L1 protein (produced at the shake-flask level). Lanes marked with “M” indicate the protein marker. Lanes marked with “+” indicate the HPV type 16 L1 protein with high purity, which was used as a positive control. Lanes marked with “P” indicate the HPV L1 protein expressed by the *E. coli* strain utilizing a plasmid-based strategy. Lanes marked with “Cgene locus” indicate the HPV L1 protein expressed by the *E. coli* strain utilizing chromosomally integrated expression strategy. (a) The HPV type 11 L1 protein was, respectively, expressed from the cassette integrated into 7 loci of the ER2566 strain. (b) L1 protein from HPV types 6, 16, 18, 31, 33, 45, 52, and 58 were, respectively, expressed from the cassette integrated into the kdsD locus of the ER2566 strain. (c) and (d) L1 protein from HPV types 18 and 52 were, respectively, expressed from the cassette integrated into 6 loci (as indicated above) of the ER2566 strain.


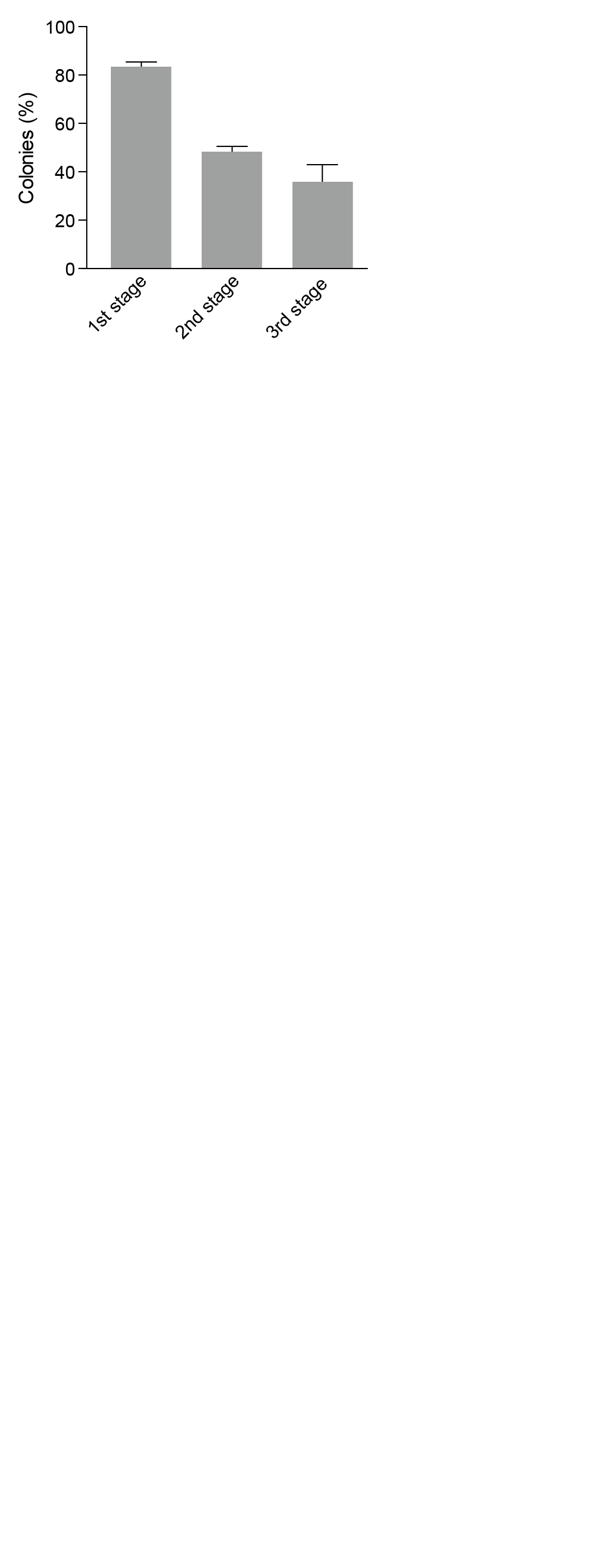


Fig. S3. Evaluation of plasmid maintenance during the three-stage fermentation process.


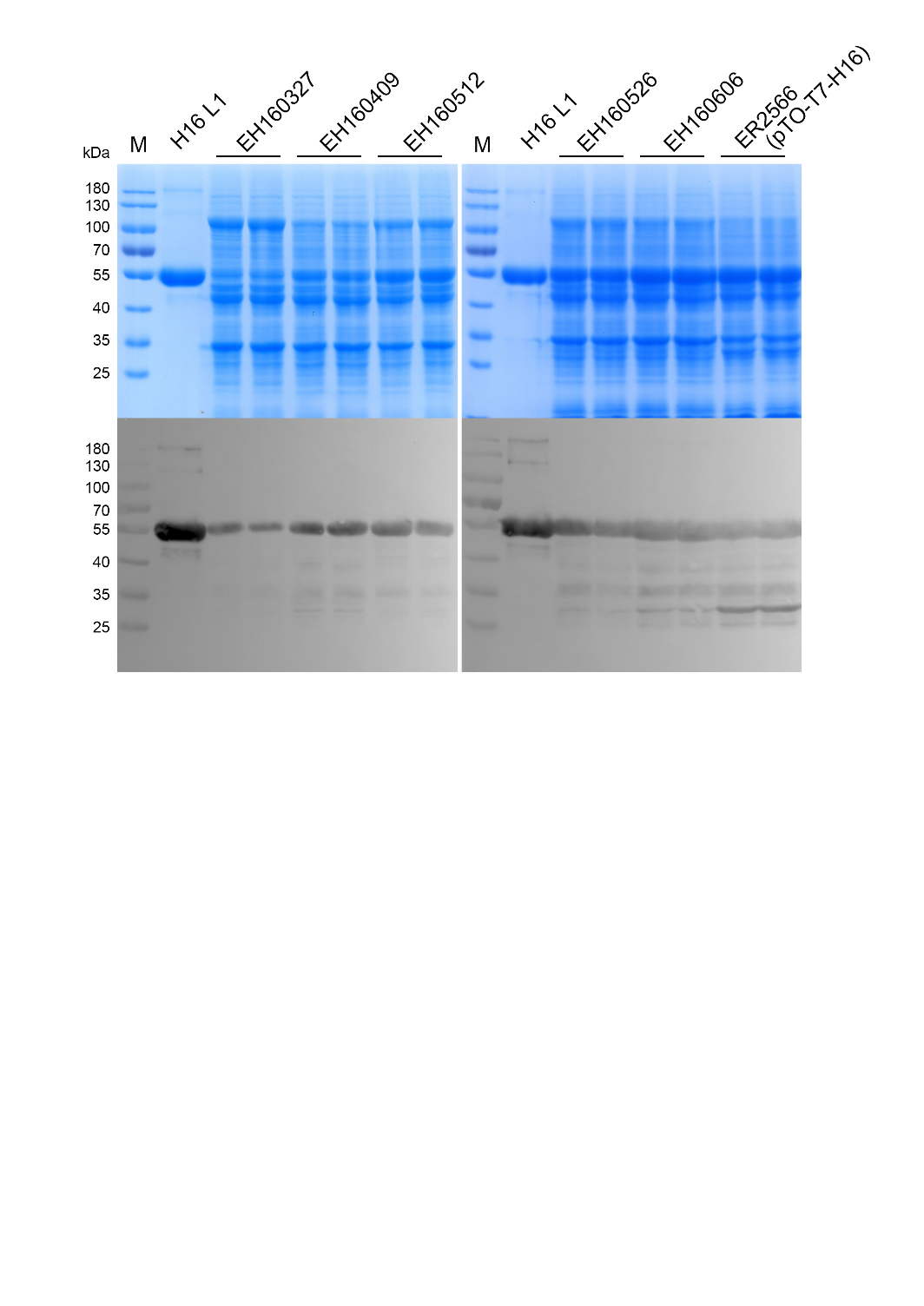


Fig. S4. SDS-PAGE and western blotting analysis of the HPV type 16 L1 protein expressed by the constructed *E. coli* strains bearing multi-copies of the integrated expression cassette. Lanes marked with “M” indicate the protein marker. Lanes marked with “+” indicate the HPV type 16 L1 protein with high purity, which was used as a positive control.


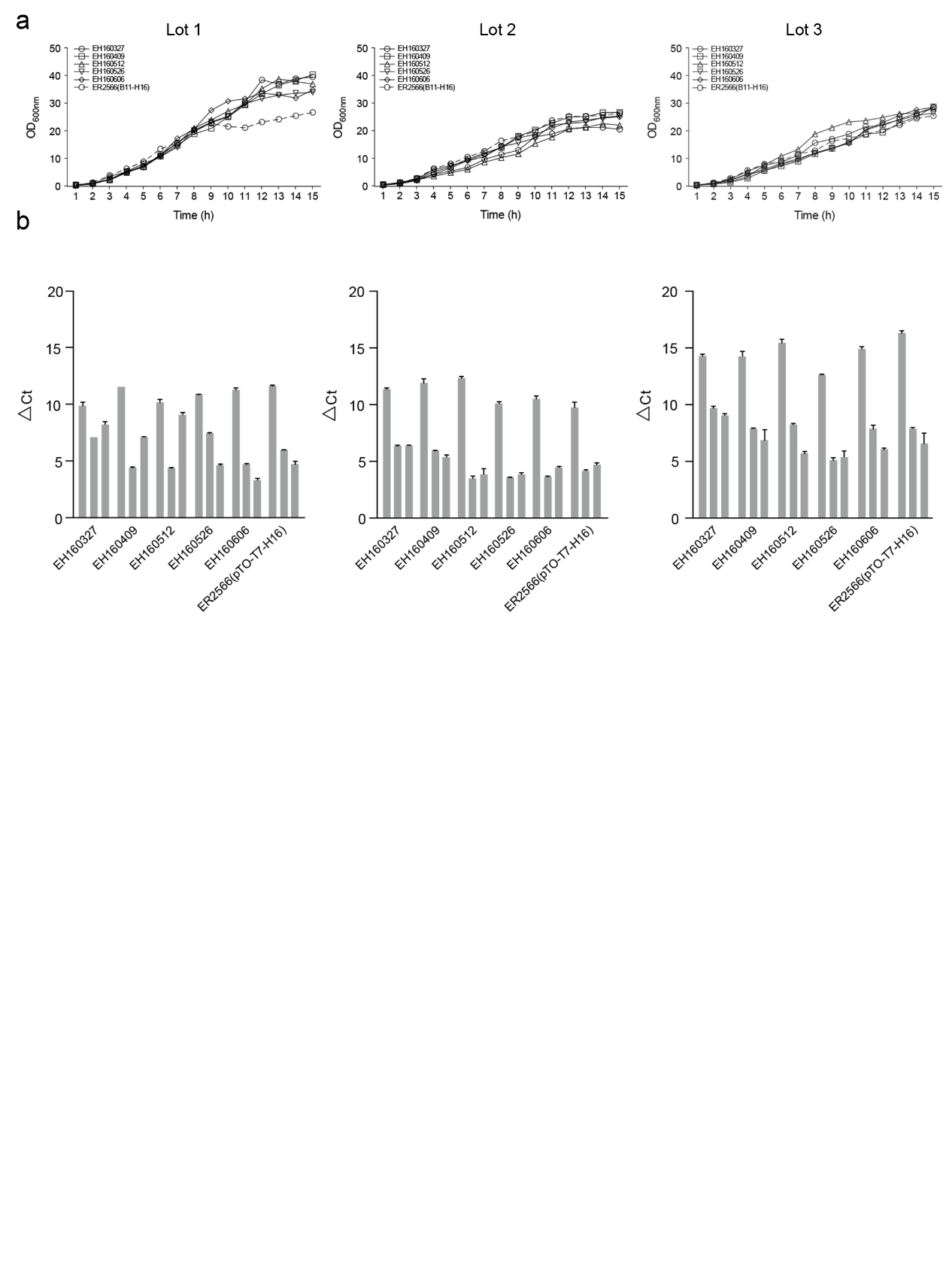


Fig. S5. Growth curves (a) and transcript levels (b) of the recombinant *E. coli* strains bearing multiple copies of the HPV16 target gene during large-scale fermentation. The time points were set as 0, 1, and 6 h post-IPTG induction.
